# Unique Flap Conformation in an HIV-1 Protease with High-Level Darunavir Resistance

**DOI:** 10.3389/fmicb.2016.00061

**Published:** 2016-02-03

**Authors:** Masaaki Nakashima, Hirotaka Ode, Koji Suzuki, Masayuki Fujino, Masami Maejima, Yuki Kimura, Takashi Masaoka, Junko Hattori, Masakazu Matsuda, Atsuko Hachiya, Yoshiyuki Yokomaku, Atsuo Suzuki, Nobuhisa Watanabe, Wataru Sugiura, Yasumasa Iwatani

**Affiliations:** ^1^Department of Infectious Diseases and Immunology, Clinical Research Center, National Hospital Organization Nagoya Medical CenterNagoya, Japan; ^2^Department of Biotechnology, Nagoya University Graduate School of EngineeringNagoya, Japan; ^3^AIDS Research Center, National Institute of Infectious DiseasesTokyo, Japan; ^4^Synchrotron Radiation Research Center, Nagoya UniversityNagoya, Japan; ^5^Department of AIDS Research, Nagoya University Graduate School of MedicineNagoya, Japan

**Keywords:** Darunavir, HIV-1 protease, drug resistance, crystal structure, flap, I50V, protease inhibitor, molecular dynamics simulation

## Abstract

Darunavir (DRV) is one of the most powerful protease inhibitors (PIs) for treating human immunodeficiency virus type-1 (HIV-1) infection and presents a high genetic barrier to the generation of resistant viruses. However, DRV-resistant HIV-1 infrequently emerges from viruses exhibiting resistance to other protease inhibitors. To address this resistance, researchers have gathered genetic information on DRV resistance. In contrast, few structural insights into the mechanism underlying DRV resistance are available. To elucidate this mechanism, we determined the crystal structure of the ligand-free state of a protease with high-level DRV resistance and six DRV resistance-associated mutations (including I47V and I50V), which we generated by *in vitro* selection. This crystal structure showed a unique curling conformation at the flap regions that was not found in the previously reported ligand-free protease structures. Molecular dynamics simulations indicated that the curled flap conformation altered the flap dynamics. These results suggest that the preference for a unique flap conformation influences DRV binding. These results provide new structural insights into elucidating the molecular mechanism of DRV resistance and aid to develop PIs effective against DRV-resistant viruses.

## Introduction

At present, more than 20 antiviral drugs are clinically available to treat patients infected with human immunodeficiency virus type 1 (HIV-1). PIs, such as darunavir (DRV; previously known as TMC114), suppress HIV-1 replication by inhibiting the functions of the viral protease (PR). DRV is a second-generation HIV-1 PI and is one of the most potent anti-HIV-1 drugs, owing to its high antiviral activity and high genetic barrier to the generation of resistant viruses (Koh et al., 2003; De Meyer et al., 2005; Surleraux et al., 2005; Ghosh et al., 2006; Dierynck et al., 2007). However, a history of drug-resistant HIV infection and/or experience of treatment-failure with other regimens could raise concerns about the emergence of DRV-resistant virus, which subsequently results in incomplete viral suppression (Koh et al., 2010; Dierynck et al., 2011). The increasing number of these DRV resistance-associated mutations at baseline raises the risk of developing DRV resistance (Delaugerre et al., 2008). The latest International AIDS Society (IAS)-USA panel list shows 11 mutations associated with DRV resistance: V11I, V32I, L33F, I47V, I50V, I54M/L, T74P, L76V, I84V, and L89V (Wensing et al., 2014). The emergence of DRV-resistant viruses is the most troublesome clinical issue.

The mechanisms underlying DRV resistance must be elucidated to overcome DRV resistance and develop active drugs against DRV-resistant viruses. One valuable approach is to obtain three-dimensional (3D) structure information of PR variants with high-level resistance to DRV. Nonetheless, few reports have published 3D structures of PR variants with high-level resistance to DRV (Saskova et al., 2009; Agniswamy et al., 2012; Zhang et al., 2014), probably because of the rarity of these PRs. In this study, we generated viruses with high-level resistance to DRV by *in vitro* selection and determined a crystal structure of an HIV-1 PR with high-level resistance to DRV. We obtained a variant with high-level resistance to DRV, which carries I47V and I50V in the PR region. These two mutations are known as the major DRV-resistance mutations (Wensing et al., 2014), although it has also been reported that they reduce viral PR activity and viral fitness (Pazhanisamy et al., 1996; Maguire et al., 2002; Prado et al., 2002; Liu et al., 2005). The solved high-resolution crystal structure of the viral PR exhibited a unique curling conformation at the flap regions (residues 43–58) (Hornak et al., 2006b) that was not found in the previously reported PR structures. These results provide new structural insights into elucidating the molecular mechanism of DRV resistance and aid to develop PIs effective against DRV-resistant viruses.

## Materials and methods

### Sample collection

Twenty samples with viral sequences that implied resistance to multiple drugs were selected from patient samples sent to the Japanese Drug Resistance HIV-1 Surveillance Network for regular drug-resistance testing from January 2005 to December 2007 (Table 1; Hattori et al., 2010). This study was conducted according to the principles of the Declaration of Helsinki. The Ethical Committee at the National Institute of Infectious Diseases approved the study. All patients provided written informed consent for the collection of samples and the subsequent analyses.

**Table 1:** **Twenty multi-drug resistant HIV-1 isolates from clinical samples selected in this study**.

**Patient ID**	**Exposed PIs**	**Genotypic resistance mutations**		
		**Major**	**Minor**	Other^*^
FS492	SQV, IDV	I54V, V82F, L90M	G16E, K20I, I62V, L63P	8
FS796	IDV, NFV	M46I, V82F, L90M	L63P, H69K	3
FS1041	RTV, NFV	V32I, I54V, V82M, L90M	K20R, K43T, M36I, I62V, L63P, A71V, G73S	3
FS1120	SQV, RTV	I54V, V82S, L90M	L10I, L33F, Q58E, L63P, A71V, G73S	2
FS1182	SQV, NFV	L90M	K20KM, L10I, L63P, A71IV, V77I, G73S	3
FS1673	SQV, RTV, IDV, NFV	M46I, I84V, L90M	K20KT, I62IV, L63P, A71V, G73S, V77I	7
FS1745	SQV, RTV, IDV, NFV	M46I, I84V, L90M	L10I, M36I, I62V, L63P, A71T, G73T	7
FS1762	RTV, DV, NFV	M46I, I54V, V82A	L10I, L24I, L33I, M36L, K43T, I62V, L63P, I64IV	3
FS1777	NFV	M46MI, L90LM	L10LI, I62V, L63P, A71AV, G73GS, V77I	4
FS2510	SQV, RTV, NFV	D30N, M46I, I54V, N88D, L90M	L10I, L23I, K43T, I62V, L63P, A71T, V77I	5
FS2628	SQV, RTV, IDV, NFV	M46I, I84V, L90M	L10I, K20I, M36V, I62V, L63P, A71V, G73S	3
FS2699	RTV, NFV	I54V, V82A, L90M	L10I, K20I, M36I, A71V, G73S	6
FS2715	IDV, NFV	I54V, V82A, L90M	L10I, M36V, I62V, L63P, A71V, G73T	2
FS2735	SQV, RTV, IDV, NFV	M46MI, I84IV, L90LM	L10LI, I62IV, L63P, A71V, G73GS, V77VI	8
FS5929^†^	SQV, RTV, IDV, NFV, APV, LPV	M46I, I54V, V82F, L90M	L10I, L23I, L33F, F53L, Q58E, I62IV, L63P, A71V, G73S, V77I	3
FS6174	SQV, RTV, NFV	D30N, I54V, N88D, L90M	L10V, K20T, M36I, I62V, L63T, I64IV, A71V	9
FS6175	SQV, RTV, LPV, ATV	V32I, M46L, I54V, V82A, L90M	L10V, K20R, M36I, K43KT, F53FL, I62IV, L63P, A71V	4
FS6189	NFV	D30N, N88D, L90M	L63P, A71I, V77I	4
FS6190	SQV, RTV, NFV, LPV, ATV	M46I, I84V, L90M	L10I, K20T, M36I, F53L, Q58QE, I62V, L63AT, A71V	8
FS7766	RTV, APV, ATV	L90M	L10I, L63P, V77I	3

* *The column shows the number of PR mutations in each patient other than major and minor mutations*.

† *The multiple drug-resistant virus sample used in this study to induce DRV-resistant HIV-1*.

### *In vitro* selection of a DRV-resistant virus

We infected each virus derived from the patient serum into the R5-MaRBLE cell line (Chiba-Mizutani et al., 2007) and induced resistance by treating with 2 nM DRV. The cultures were maintained by changing half of the medium every 3–5 days and by step-wise increases in the DRV concentration to 1000 nM.

### *In vitro* phenotype assay to examine drug susceptibility

The susceptibilities to the PIs were evaluated using an in-house drug susceptibility assay with the R5-MaRBLE cell line as described elsewhere (Chiba-Mizutani et al., 2007; Shibata et al., 2011). Inhibitory concentration 50% (IC_50_) values were obtained from three independent experiments.

### Extraction and amplification of viral RNA

Viral RNA was extracted from the cultured system as follows. First, virus particles in the cell culture supernatant were collected by centrifugation at 20,000 × g at 4°C for 1.5 h. The collected particles were suspended in 300 μL of RNAgents Denaturing Solution (Promega, Madison, WI, USA). Then, the RNA was purified by phenol-chloroform extraction. The *gag-PR* region (625–3402; positions based on HXB2 numbering) of the purified RNA was reverse transcribed using a PrimeScript II High Fidelity One Step RT-PCR Kit (Takara Bio Inc., Kusatsu, Japan). Subsequently, an inner *gag-PR* region (681–3348) was amplified by nested PCR using PrimeSTAR GXL DNA Polymerase (Takara Bio Inc.). The primer sets used for the amplification were as follows: reverse transcription PCR, 5′-ATCTCTAGCAGTGGC GCCCGAACAG and 5′-TAC TTCTGTTAGTGCTTTGGTTCC and nested PCR, 5′-CTCTCTCGACGC AGGACTCG and 5′-TAA TCCCTGCATAAATCTGACTTGC.

### Construction of recombinant viruses

A DNA fragment of *gag-PR* region (699–2580) was inserted into the pNL4-3 clone vector using a GeneArt Seamless Cloning and Assembly Kit (Thermo Fisher Scientific, Waltham, MA, USA). First, we amplified the target *gag-PR* region with PrimeSTAR GXL Polymerase. The primer set used for amplification was as follows: 5′-CGGCTT GCTGAAGCGCGCACAGCAAGAGGCGAGGGGCGGCGACTG and 5′-TTA CTGGTACAGTCTCAATAGGACTAATGGG. The amplified PCR product was ligated with pNL4-3 without *gag-PR*.

### Construction of the inactive PR expression vector

The full-length *PR* region (2253–2549) was amplified by nested PCR using KOD DNA Polymerase (TOYOBO, Osaka, Japan). The primer set used for amplification was as follows: 5′-ATATACATATGCCTCAGAT CACTCTTTGG and 5′-TG GTGCTCGAGTTACTAAAAATTTAAAGTGCAGCC. Subsequently, the PCR product was inserted into pET-41a(+) (Merck Millipore, Billerica, MA, USA) using NdeI and XhoI restriction enzymes and a DNA Ligation Kit ver. 2.1 (Takara Bio Inc.). Mutagenesis was performed to obtain an inactive D25N PR mutant.

### Expression, purification, and refolding of the inactive PR

The enzymatically-inactive PR was expressed, purified, and then refolded by using a method similar to that in the previous report by Dr. Schiffer's group (King et al., 2002). Briefly, the inactive PR was expressed in LB medium with 1 mM IPTG at 37°C. The inclusion bodies with the PR were collected by using a French press and subsequently centrifuged at 10,000 × g at 4°C for 5 min. The collected inclusion bodies were washed with 2 M urea, and the PR protein was solubilized in a 50% acetic acid solution. The obtained PR protein was purified by gel filtration with a HiLoad 26/60 Superdex75 (GE Healthcare Bio-Sciences, Pittsburgh, PA, USA) and AKTAPrime (GE Healthcare Bio-Sciences). The PR was refolded in buffer containing sodium acetate (pH 5.5). Finally, the PR was further purified by gel filtration with a HiLoad 26/60 Superdex75 column (GE Healthcare Bio-Sciences).

### Crystallization of the inactive PR

The purified PR was concentrated to a final concentration of 2.6 mg mL^−1^ with a VIVASPIN MW5000 concentrator (GE Healthcare Bio-Sciences). Then, initial crystallization conditions of PR were screened using following crystallization screening kits: Wizard I (Emerald Biosystems, Bainbridge Island, WA, USA), Wizard II (Emerald Biosystems), and JCSG-*plus* Screen MD-137 (Molecular Dimensions, Suffolk, UK). The final optimized crystallization condition after iterative optimization cycles was at 0.84 M Lithium chloride, 0.84 mM Sodium citrate, 17%(w/v) PEG 6000, 10% Glycerol, and 5% Ethylene glycol. The crystal was obtained by the hanging-drop vapor-diffusion method. The drops were incubated at 20°C.

### X-ray diffraction and data collection

The X-ray diffraction data were collected at the SPring-8 beamline BL38B1 (Sayo, Hyogo, Japan). The data were integrated with the HKL2000 program (Otwinowski and Minor, 1997). The structure of the inactive PR was determined by the molecular replacement method with the MOLREP programs (Vargin and Teplyakov, 1997; Winn et al., 2011) using a wild-type (WT) PR structure (Protein Data Bank (PDB) ID: 1KJ7) (Prabu-Jeyabalan et al., 2002) as the search model. The structure was further refined with the REFMAC5 and COOT programs (Murshudov et al., 1997; Emsley et al., 2010).

### Molecular dynamics (MD) simulations

We performed 30 ns MD simulations of the ligand-free states of the PRs under explicit water conditions. The initial structures of the PRs were generated based on a known crystal structure (PDB ID: 1HHP) (Spinelli et al., 1991) and the structure solved in this study. In the initial structures, the PRs were surrounded by approximately 12,000 water molecules, and N25 was changed to the catalytically active D25. We conducted energy minimization for the PR system by first using 10,000 steps of the steepest descent method and then 10,000 steps of the conjugated gradient method. We subsequently heated the energy-minimized system to 310 K (~37°C) by using the NVT ensemble. Then, we performed MD simulations at 310 K and 1 atm by using the NPT ensemble. During the simulations, hydrogen bonds among residues in the fireman's grip, D25/T26/G27/D25′/T26′/G27′, were retained with a harmonic potential of 100 kcal mol^−1^ Å^−2^ and 100 kcal mol^−1^ degree^−2^.

We also predicted the structures of mutant PRs in complex with DRV with 6.0 ns MD simulations by using a method similar to that in our previous reports (Ode et al., 2007a,b). Briefly, the initial structure of each molecule was generated from a crystal structure of PR in complex with DRV (PDB ID: 1T3R) (Surleraux et al., 2005). We performed 6.0 ns MD simulations of these structures and then estimated the binding energies between PR and DRV by the MMPBSA method using 1000 trajectories from the well-equilibrated final 1.0 ns of the simulations. For the structural comparison, the representative structure among 1000 snapshots taken during the last 1.0 ns of the simulations was used. The formation of a hydrogen bond was defined as described in our previous studies (Ode et al., 2007a,b).

The simulations were conducted with the AMBER9 software package (Case et al., 2006). The ff10 force field (Hornak et al., 2006a) and gaff (Wang et al., 2004) were used to calculate the energies and forces in the simulations.

## Results

### *In vitro* selection yielded variants that accumulated DRV resistance-associated mutations

**Figure 1:**
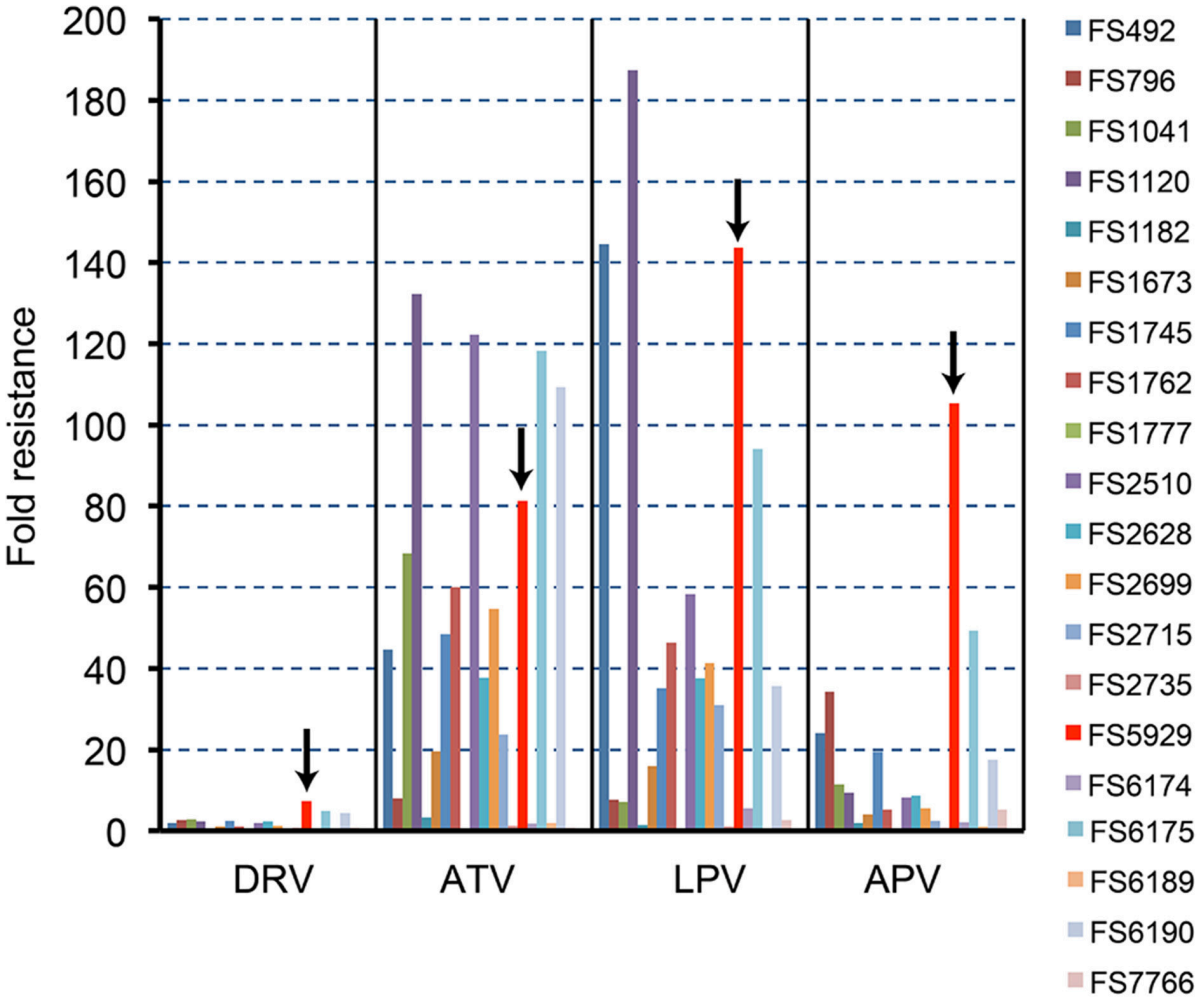
**The PI susceptibilities of 20 clinical HIV-1 isolates**. The arrows in this figure highlight the results for FS5929. The “fold resistance” in IC_50_ are given relative to the IC_50_ values for the reference wild-type HIV-1 JR-CSF, according to the equation, fold resistance = (the IC_50_ against a clinical isolate)/(the IC_50_ against the JR-CSF).

**Figure 2:**
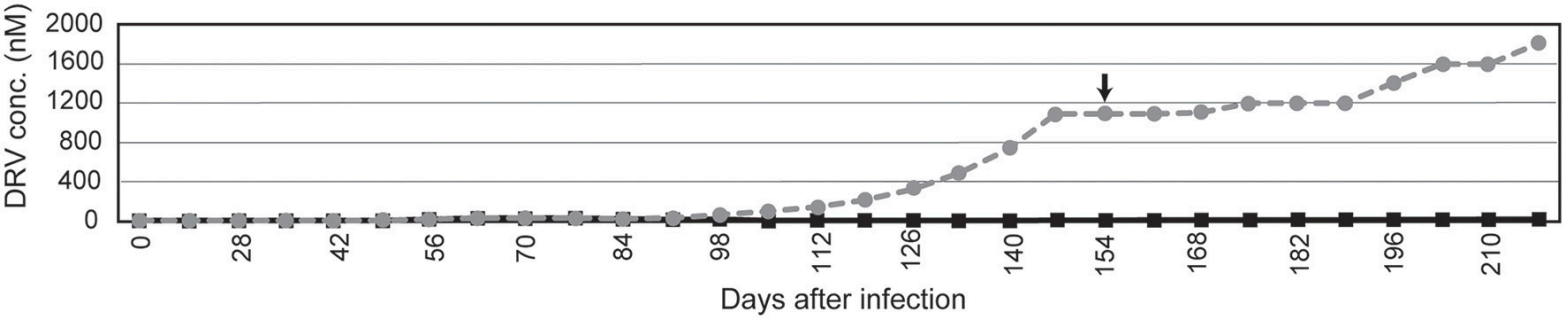
***In vitro* selection to induce DRV-resistant viruses**. The selection of FS5929 and HIV-1 JR-CSF are indicated with gray dotted and black solid lines, respectively. The arrow indicates the sampling time point for the collection of FS5929R, which we focused on in this study. The x-axis and y-axis represent “days after the initial viral infection to cell culture” and “DRV concentration (μM) in cell culture medium,” respectively.

To obtain a crystal structure of an HIV-1 PR with high-level resistance to DRV, we generated a virus with high-level resistance to DRV from a clinically isolated multiple drug-resistant sample (FS5929). FS5929 contained one minor DRV resistance mutation (L33F) (Table 2) and conferred the highest DRV resistance (7.7-fold increase compared with the WT HIV-1 JR-CSF) of the 20 multiple drug-resistant samples selected for this study (Figure 1 and Table 1). After 154 days of culture in the presence of increasing concentrations of DRV, we obtained a virus mixture (FS5929R) with high-level resistance to DRV that was viable in cultures with 1000 nM DRV (Figure 2). Population sequence analyses of the PR region indicated that FS5929R consisted of at least seven variants (Table 2). The major virus (referred to as FS5929R1) had two major DRV resistance mutations (I47V and I50V), four minor mutations (V11I, V32I, L33F, and L89V), and V82F. The other minor variants had I47V mutation and either of I50V or L76V mutation although the other sequences were similar to that of the major virus. Additionally, we examined the PR cleavage sites in the *gag* sequences. No sequence changes around the cleavage sites occurred during culture (Table 3), although mutations at the flanking regions of the cleavage sites were observed in both FS5929 and FS5929R1 (except for the sites between the capsid and p2 and between p1 and p6). Together, these results show that the high-level DRV resistance of FS5929R is mainly attributed to the accumulation of DRV resistance-associated mutations in the PR region. In contrast, no DRV-resistant virus was developed by the induction of the WT HIV-1 JR-CSF for over half of a year (Figure 2) as detailed in previous reports (De Meyer et al., 2005; Surleraux et al., 2005; Dierynck et al., 2007).

**Table 2:** **List of PR mutations detected during *in vitro* selection**.

		N^*^	**DRV resistance mutations**							**Other^†^**			
			**Major^†^**			**Minor^†^**				
			**47**	**50**	**76**	**11**	**32**	**33**	**89**	**54**	**60**	**62**	**82**
NL4-3			I	I	L	V	V	L	L	I	D	I	V
FS5929	(Day 0)^‡^		–	–	–	–	–	F	–	V	–	V	F
FS5929R	(Day 154)^‡^	4$	V	V	–	I	I	F	V	V	E	–	F
		3	V	V	–	–	I	F	V	V	E	–	F
		2	V	V	–	I	I	F	V	V	E	–	L
		1	V	–	V	I	I	F	V	V	E	–	F
		1	V	–	V	–	I	F	V	V	E	–	F
		1	V	–	–	I	I	F	V	A	E	–	F
		1	V	–	–	I	I	F	V	V	E	–	F

* *The number of clones with the combination of mutations*.

† *Classification of the IAS-USA mutation list at June/July 2014*.

‡ *FS5929 and FS5929R denote the input virus for the in vitro selection (day 0) and the virus mixture obtained at the day 154 in this study*.

*The sequence contains mutations: L10I, L23I, L33F, M46I, F53L, I54V, G57R, Q58E, I62V, L63P, H69R, A71V, G73S, V77I, V82F, L90M, and I93L, compared with NL4-3. L33F is reported to be associated with DRV-resistance mutations*.

$ *The major virus in the virus mixture obtained at the day 154 (FS5929R) was defined as FS5929R1*.

**Table 3:** **Gag sequence of major DRV-resistant viruses**.

Positions*		10	20	30	40	50	60
NL4-3		MGARASVLSG	GELDKWEKIR	LRPGGKKQYK	LKHIVWASRE	LERFAVNPGL	LETSEGCRQI
FS5929	(Day 0)	----------	----------	-------K-R	----------	----------	----------
FS5929R	(Day 154)†	----------	-K--------	-------K-R	----------	----------	----------
Positions		70	80	90	100	110	120
NL4-3		LGQLQPSLQT	GSEELRSLYN	TIAVLYCVHQ	RIDVKDTKEA	LDKIEEEQNK	SKKKAQQAAA
FS5929	(Day 0)	----------	----------	---T------	----------	-E--------	----------
FS5929R	(Day 154)	----------	----------	---T------	----------	-E--------	----------
Positions		129	139	149	159	169	179
		X	XXXXXXX				
NL4-3		DTGNNS QVS	QNYPIVQNLQ	GQMVHQAISP	RTLNAWVKVV	EEKAFSPEVI	PMFSALSEGA
FS5929	(Day 0)	------SK--	--F-------	----------	---------I	----------	----------
FS5929R	(Day 154)	------SK--	--F-------	----------	---------I	----------	----------
Positions		189	199	209	219	229	239
NL4-3		TPQDLNTMLN	TVGGHQAAMQ	MLKETINEEA	AEWDRLHPVH	AGPIAPGQMR	EPRGSDIAGT
FS5929	(Day 0)	----------	----------	----------	--------P Q	----------	---------A
FS5929R	(Day 154)	----------	----------	----------	--------P Q	----------	---------A
Positions		249	259	269	279	289	299
NL4-3		TSTLQEQIGW	MTHNPPIPVG	EIYKRWIILG	LNKIVRMYSP	TSILDIRQGP	KEPFRDYVDR
FS5929	(Day 0)	----------	--S---V---	----------	----------	V---------	----------
FS5929R	(Day 154)	----------	--N-------	----------	----------	V---------	----------
Positions		309	319	329	339	349	359
NL4-3		FYKTLRAEQA	SQEVKNWMTE	TLLVQNANPD	CKTILKALGP	GATLEEMMTA	CQGVGGPGHK
FS5929	(Day 0)	----------	----------	----------	----------	A---------	----------
FS5929R	(Day 154)	----------	----------	----------	----------	A---------	----------
Positions		369	379	389	399	409	419
		XXXXXXXX	XXXXXX	XX			
NL4-3		ARVLAEAMSQ	VTNPATIMIQ	KGNFRNQRKT	VKCFNCGKEG	HIAKNCRAPR	KKGCWKCGKE
FS5929	(Day 0)	---------L	--H-----M-	------P---	----------	-V-R------	-R--------
FS5929R	(Day 154)	---------L	--H-----M-	------P---	----------	-V-R------	----------
Positions		429	439	449	458	456	466
		X	XXXXXXX	XXXXXX	XX		
NL4-3		GHQMKDCTER	QANFLGKIWP	SHKGRPGNFL	QSRPEPTAP	PEESFRFG	EETTTPSQKQ
FS5929	(Day 0)	-------A--	-IH--E----	----------	---A-----P	AP--------	----------
FS5929R	(Day 154)	----------	-IH--E----	----------	---A-----P	AP--------	-K--------
Positions		476					
NL4-3		EPIDKELYPL	A
FS5929	(Day 0)	--M-EG----	-
FS5929R	(Day 154)	----EG----	-

*FS5929 and FS5929R denote the input viruses for the in vitro selection (day 0) and the virus mixture obtained at the day 154, respectively*.

* The eight residues flanking at each of five PR cleavage sites in Gag (MA-CA, CA-p2, p2-NC, NC-p1, and p1-p6) were highlighted with “X.”

† *The Gag sequence was shown as a major population sequence*.

### The major PR obtained from *in vitro* selection is resistant to DRV but not to TPV

Next, we focused on FS5929R1 and analyzed its resistance to DRV. We generated recombinant viruses carrying the *gag-PR* of FS5929R1 (rFS5929R1) and FS5929 (rFS5929) based on the HIV-1 NL4-3 backbone. The susceptibility assays showed that rFS5929R1 displayed a >175-fold increase in DRV resistance compared with that of WT NL4-3, whereas FS5929 was susceptible to DRV (1.9-fold increase) (Table 4). We also constructed three recombinant rFS5929R1 viruses lacking the major DRV resistance mutations I47V and/or I50V to assess the contribution of the major mutations to DRV resistance. Each rFS5929R1 virus without I47V (rFS5929R1_I47_) and rFS5929R1 without I50V (rFS5929R1_I50_) exhibited a >175-fold and 96.9-fold increase in DRV resistance, respectively, whereas the rFS5929R1 without the two mutations (rFS5929R1_I47∕I50_) exhibited a 20.4-fold increase. The results suggest that the two major mutations, especially I50V, largely contribute to the DRV resistance in rFS5929R1.

**Table 4:** **PI susceptibility assays of recombinant viruses**.

	**NL4-3**	**rFS5929**	**rFS5929R1**	**rFS5929R1_I47_**	**rFS5929R1_I50_**	**rFS5929R1_I47∕I50_**
DRV	0.0057 ± 0.0006*	0.0111 ± 0.0036 (1.9†)	>1 (>175.3)	>1 (>175.3)	0.5528 ± 0.0722 (96.9)	0.1162 ± 0.0149 (20.4)
APV	0.0278 ± 0.0042	0.4440 ± 0.1263 (16.0)	>1 (>35.9)	>1 (>35.9)	>1 (>35.9)	>1 (>35.9)
LPV	0.0182 ± 0.0011	>1 (>54.9)	>1 (>54.9)	>1 (>54.9)	>1 (>54.9)	0.9493 ± 0.0453 (52.1)
RTV	0.0347 ± 0.0049	>1 (>28.8)	>1 (>28.8)	>1 (>28.8)	>1 (>28.8)	>1 (28.8)
NFV	0.0120 ± 0.0006	>1 (>83.1)	>1 (>83.1)	>1 (>83.1)	>1 (>83.1)	>1 (>83.1)
TPV	0.1368 ± 0.0119	0.0646 ± 0.0192 (0.5)	0.0500 ± 0.0119 (0.4)	0.0204 ± 0.0068 (0.1)	0.2397 ± 0.0250 (1.8)	0.1810 ± 0.0037 (1.3)
ATV	0.0047 ± 0.0013	0.0481 ± 0.0129 (10.2)	0.0178 ± 0.0039 (3.8)	0.0490 ± 0.0109 (10.4)	0.0489 ± 0.0143 (10.4)	0.1350 ± 0.0236 (28.7)
SQV	0.0203 ± 0.0077	0.3030 ± 0.0622 (14.9)	0.2500 ± 0.0506 (12.3)	0.2631 ± 0.0555 (13.0)	0.1838 ± 0.0451 (9.1)	0.2440 ± 0.0183 (12.0)

* *IC_50_ (μM)*.

† *Fold changes from IC_50_ of HIV-1 NL4-3 are written in parentheses*.

We also measured the resistance to seven other PIs: amprenavir (APV), lopinavir (LPV), ritonavir (RTV), nelfinavir (NFV), tipranavir (TPV), atazanavir (ATV), and saquinavir (SQV). The rFS5929 and rFS5929R1 viruses exhibited 16.0- and >35.9-fold resistance, respectively, to the DRV-analog APV. Both viruses exhibited high-level resistance to LPV (>54.9 fold), RTV (>28.8 fold), and NFV (>83.1 fold). Interestingly, both viruses were susceptible to TPV (< 0.5 fold) and exhibited 3- to 15-fold resistance to ATV and SQV, suggesting that the mutations induced by the *in vitro* selection with DRV scarcely affected the susceptibilities to TPV, ATV, and SQV.

### Determination of the high-resolution crystal structure of the ligand-free state of FS5929R1 PR

**Figure 3:**
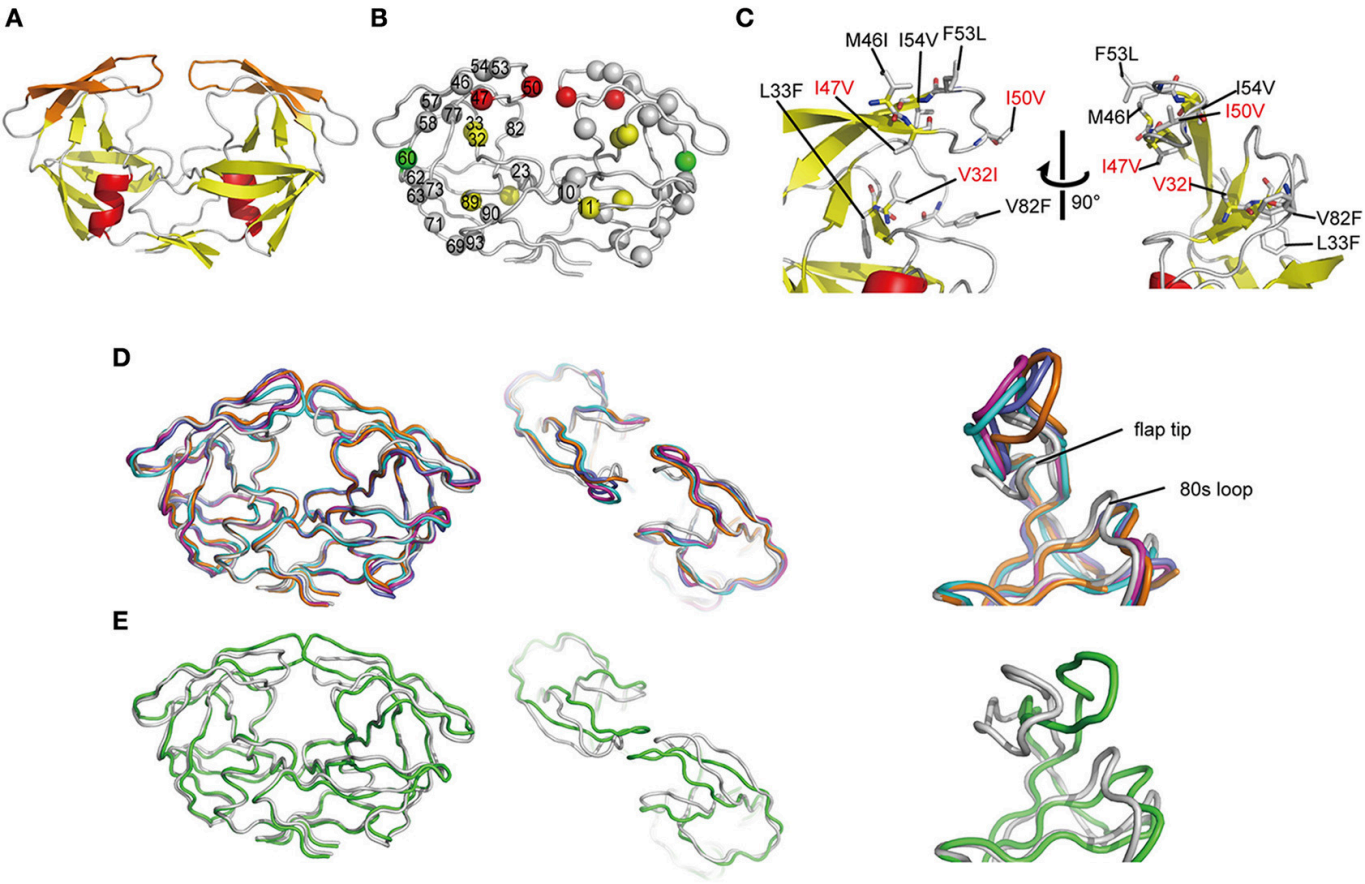
**Crystal structure of FS5929R1 PR**. **(A)** Ribbon diagram of the PR, which forms a homodimer. The α-helices and β-strands are colored in red and yellow, respectively. The flap regions (residues 43–58) are highlighted in orange. **(B)** Locations of mutated residues on the PR. The red, yellow, and green spheres indicate the locations of the major DRV resistance, minor DRV resistance, and other mutations, respectively, that appeared after the DRV-resistance induction experiment using FS5929. Mutations that originally existed in FS5929 are shown in gray spheres. **(C)** Highlighted structure around the flaps in the PR. The mutations are shown as sticks. The mutations V32I, I47V, and I50V, which appeared during the *in vitro* selection, are highlighted with red letters. **(D)** Superposition of our crystal structures of the PRs (gray) with the crystal structures of the open form [PDB IDs: 2PC0 (cyan), 1TW7 (purple), 4NPU (orange), and 3UF3 (navy)]. **(E)** Superposition of our crystal structures of the PRs with a crystal structure of the semi-open form [PDB IDs: 1HHP (green)].

**Figure 4:**
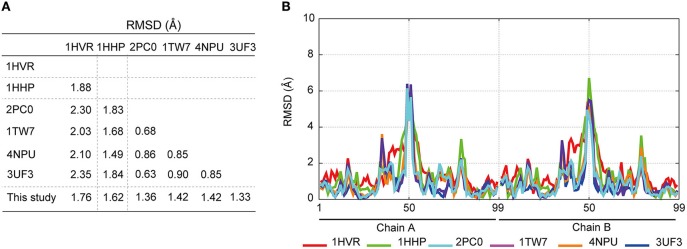
**Comparison of the crystal structures of ligand-free PRs; closed-form (PDB ID: 1HVR), semi-open form (1HHP), open form (2PC0, 1TW7, 4NPU, and 3UF3), and the PR purified in this study**. **(A)** RMSD calculations comparing the crystal structures by using the coordinates of the Cα atoms. **(B)** Comparison of each Cα atom in the PR structures with the corresponding Cα atom in the FS5929R1 PR.

To investigate the mechanism underlying the DRV resistance of FS5929R1, we determined the crystal structure of the ligand-free state of the inactive FS5929R1 PR homodimer at a 1.8 Å resolution (Figure 3 and Table 5). Our crystal structure had a conformation similar to that of the open forms (Figure 3D), as seen in the previous crystal structures of ligand-free PRs (PDB IDs: 2PC0, 1TW7, 4NPU, and 3UF3; Martin et al., 2005; Heaslet et al., 2007; Agniswamy et al., 2012; Zhang et al., 2014). Although two additional PR conformations have been reported [a semi-open form favored by the ligand-free state of the WT PR (PDB ID: 1HHP) (Spinelli et al., 1991; Hornak et al., 2006b; Deng et al., 2011) and a closed form favored by the ligand-bound state of PR (e.g., PDB ID: 1HVR) (Lam et al., 1994)], few contacts existed between the flap regions of the two monomers in our structure, which was in contrast to these semi-open or closed forms. The root mean squared deviation (RMSD) values of all Cα atoms in our crystal structure compared with those in the open-form structures (2PC0, 1TW7, 4NPU, and 3UF3) were 1.36, 1.42, 1.42, and 1.33 Å, respectively (Figure 4). Contrastingly, the crystal structures of the semi-open and closed forms (1HHP and 1HVR) showed higher RMSD values (1.62 and 1.76 Å) than for the open-form structures (Figure 3E). Notably, two of the open-form structures (4NPU and 3UF3) were the previously reported crystal structures of other PRs with high-level resistance to DRV, suggesting that PRs with high-level resistance to DRV commonly favor configurations that resemble the open form.

**Table 5:** **Crystallographic data collection and refinement statistics**.

**Parameter**	**Value(s) for HIV-1 PR**
**DATA COLLECTION**	
Space group	*P*1
Unit cell dimensions	
*a* (Å)	37.1
*b* (Å)	48.7
*c* (Å)	54.2
α (degree)	89.9
β (degree)	74.0
γ (degree)	86.7
Wave length (Å)	1.0
Resolution (Å)	50–1.80 (1.86–1.80)
*R*_merge_ (%)	4.4 (19.4)
Average *I*/σ (I)	26.9 (3.7)
Completeness (%)	96.0 (92.7)
Multiplicity	1.9 (1.9)
**REFINEMENT**	
Unique reflections	30928
*R*_work_ (%)/*R*_free_ (%)	19.6/24.0
No. of non-H atoms	
Protein	3174
Chloride ion	5
Water molecules	142
RMS deviation from ideality	
Bonds (Å)	0.019
Angle distance (degree)	2.06
Ramachandran plot	
Preferred region (%)	95.2
Allowed region (%)	4.0
Outlier regions (%)	0.8
Average B-factors (Å^2^)	
Main chain	25.3
Side chain	28.0
Solvent	33.9

*Values in parentheses correspond to the highest resolution shell. The structure has been deposited as 5B18 in Protein Data Bank (PDB)*.

Our crystal structure had a distinct conformation at the flap regions and the 80s loops that was absent in the open-form structures. The flap tip that are around the 50th residue curled inward in our crystal structure, whereas the 80s loops were slightly shifted inside of the PR. Therefore, the flap tips approached the 80s loop within the same monomer. The distance between the Cα atoms of the 50th and 80th residues within the same monomer of our crystal structure was approximately 8.9 Å, whereas the distances were approximately 14.1 and 14.0 Å in the open-form crystal structures (4NPU and 3UF3, respectively). Interestingly, the regions included the two major DRV resistance mutations (I47V and I50V) and one minor mutation (V32I), which were induced by the *in vitro* selection (Figure 3C). Hence, the unique curling structure at the flap regions was likely associated with the DRV resistance of FS5929R1.

**Figure 5:**
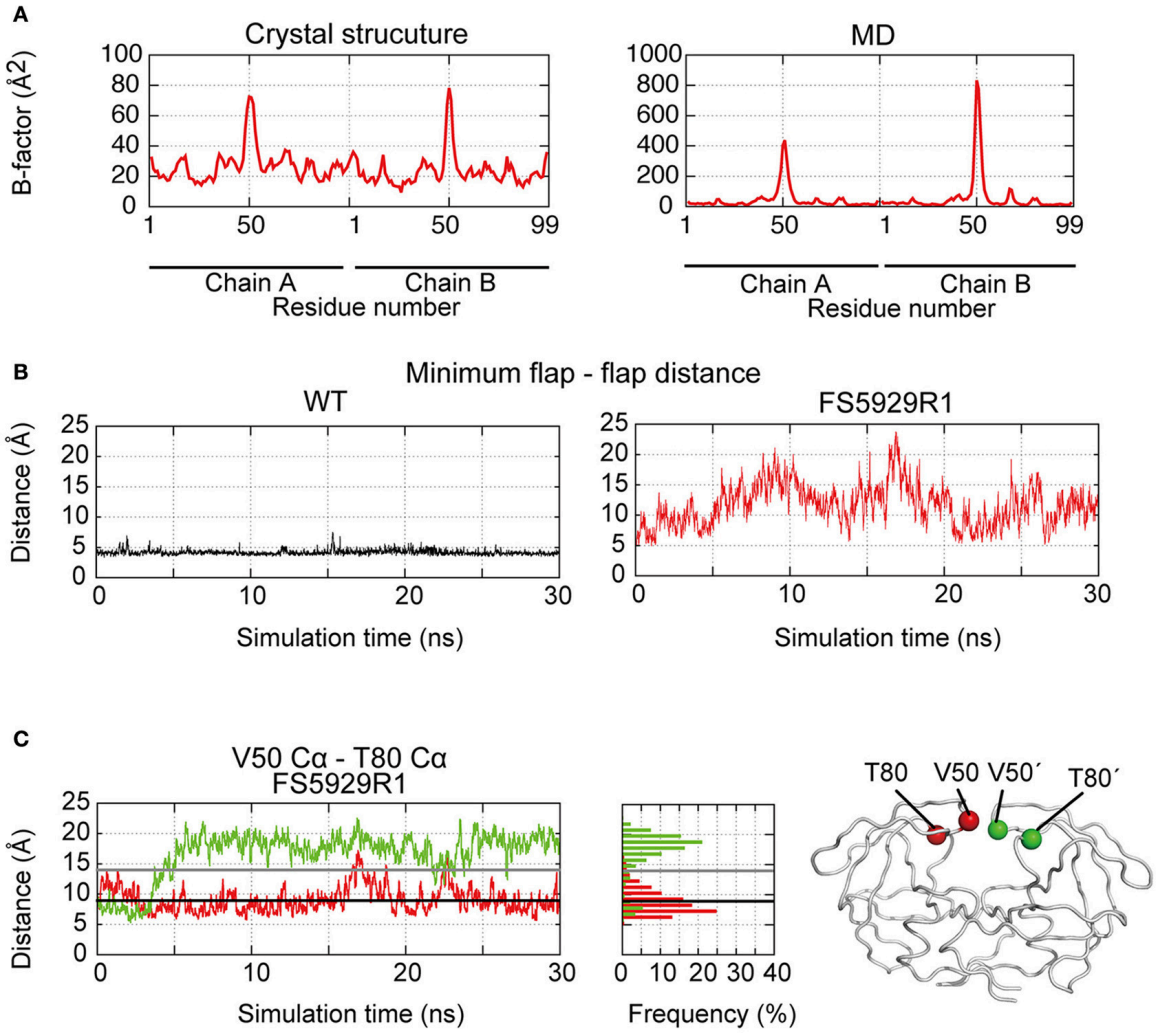
**Flap dynamics in the crystal structure and MD simulations**. **(A)** B factors of the respective residues in the FS5929R1 PR estimated from the crystal structure and MD simulations. **(B)** The minimum distance between two flaps in the WT and FS5929R1 PRs calculated with an in-house program. **(C)** The time course of the distance between the V50 Cα and the T80 Cα (left, red) or between the V50′ Cα and the T80′ Cα (left, green) and their histogram (center). The black and gray solid lines indicate the distances in the crystal structure from our study and an open-form structure (PDB ID: 3UF3), respectively. The structural positions of the V50 and T80 Cαs (red) in one monomer and the V50′ and T80′ Cαs in a second monomer are highlighted on the right side of the figure of the PR structure.

To assess the stability of the unique curling conformation at the mobile flap regions of the FS5929R1 PR (Figure 5A), we performed 30.0 ns-timescale MD simulations using the determined structure as the initial structure. The MD simulations suggested that the FS5929R1 PR favored conformations in which the flaps in each monomer were separated, which was in contrast to the WT PR (Figure 5B and Supplemental Movies S1, S2). Furthermore, constant curling at the flap region was observed in one monomer but not the second monomer (Figure 5C). These observations suggested that the flap in at least one monomer would frequently adopt the curling conformation despite the high mobility of the flap.

### Structure prediction of the FS5929R1 PR in complex with DRV

**Figure 6:**
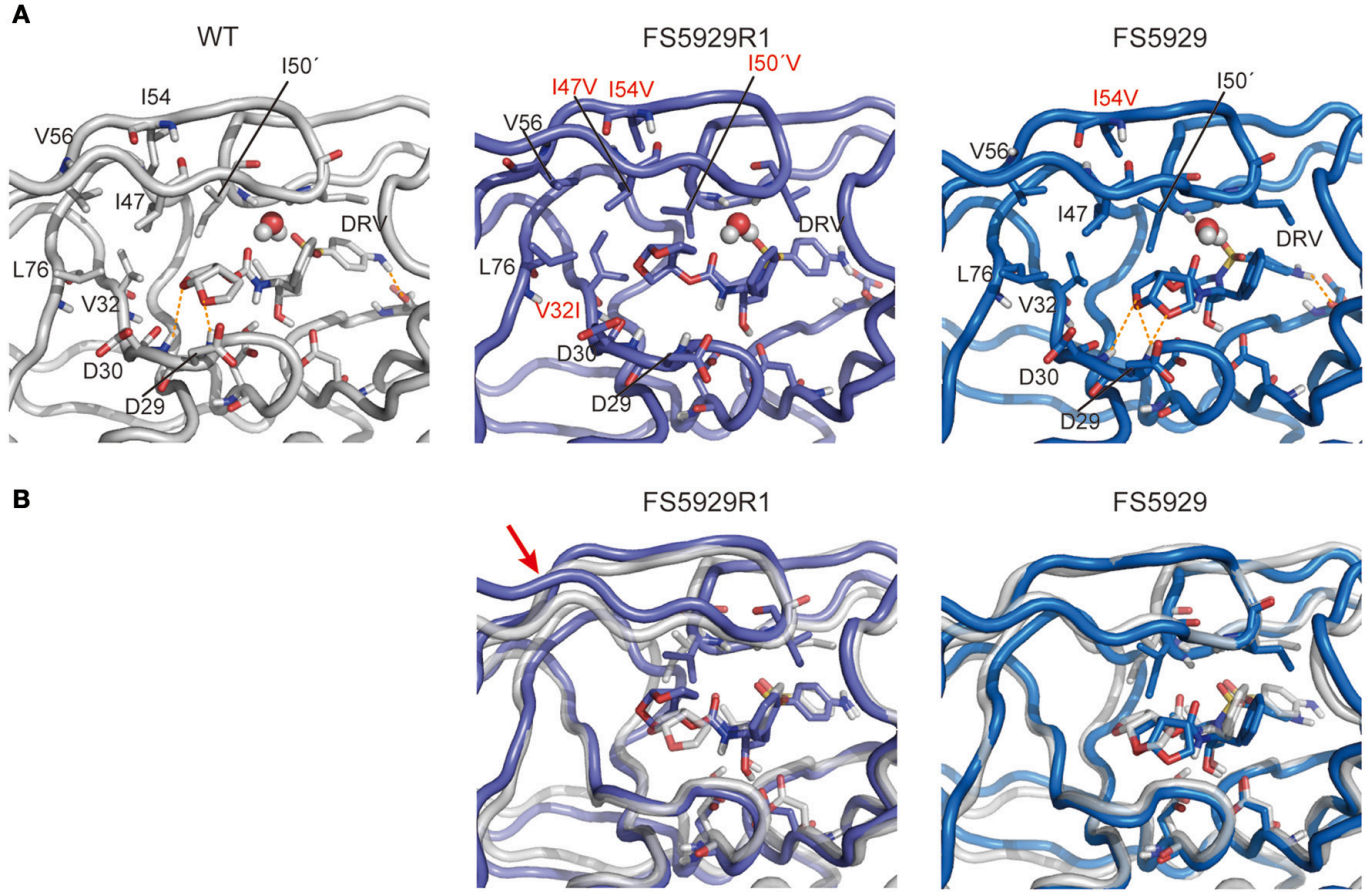
**Representative structures of simulations of each PR with DRV**. **(A)** Interactions between PR and DRV. The orange dotted lines indicate hydrogen bonds. **(B)** Superposition onto the WT PR structure. Stick representations show important residues in WT (gray), FS5929R1 (purple), and FS5929 PR (blue) structures. The red arrow indicates the flap region shifted outward in the FS5929R1 PR with DRV.

**Figure 7:**
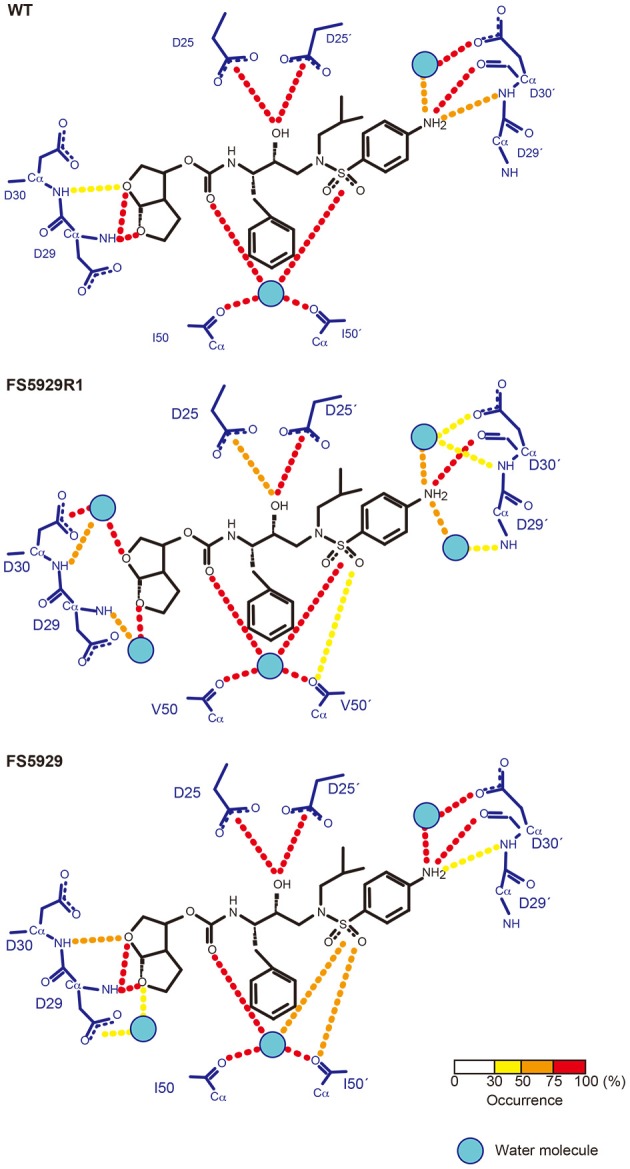
**Hydrogen bond network between PR and DRV predicted from MD simulations**. The residues in PR and DRV are highlighted with blue and black, respectively. Hydrogen bonds are represented by dotted lines colored according to their occurrence during the 5.0–6.0 ns simulations, as shown in the bottom bar.

Finally, to evaluate the binding mode of DRV to the FS5929R and FS5929R1 PRs, we predicted the interactions of both PRs with DRV, from a crystal structure of the WT PR in complex with DRV, by MD simulations (Figure 6) as described in our previous reports (Ode et al., 2007a,b). The MD simulations indicated that a flap region of FS5929R1 PR in complex with DRV shifts outward compared with that of the WT PR in complex with DRV; this change was not observed in FS5929 PR in complex with DRV. Furthermore, the FS5929R1 PR could not create direct hydrogen bonds with the DRV *bis*-tetrahydrofuran (*bis*-THF) moiety and the central hydroxyl group (Figure 7). In contrast, FS5929 PR could create hydrogen bonds with DRV that were similar to those of the WT PR despite slight differences in the PR-DRV hydrogen bond networks between the WT and FS5929 PRs. The binding energy calculations indicated a 10.9 kcal mol^−1^ loss of the binding energy with DRV for each FS5929R1 PR and a 0.8 kcal mol^−1^ loss for FS5929 PR compared with the WT PR in complex with DRV. These results were consistent with the DRV susceptibility results shown in Table 4, suggesting that the conformational change in the flap likely affected the binding of DRV to PR.

## Discussion

The virus with high-level resistance to DRV that was generated in this study (referred to as FS5929R1) harbored 6 DRV resistance-associated mutations in its PR region. The drug susceptibility tests with the recombinant virus variants suggested that the *in vitro* selection specifically increased the resistance to DRV and its structural analog APV (Table 4). The two major mutations (especially I50V) greatly contributed to the resistance of the PR, which was in agreement with the effects of single substitutions on the binding of DRV to the WT PR (De Meyer et al., 2006; Tremblay, 2008).

Notably, the crystal structure of the ligand-free FS5929R1 PR showed an open-form configuration that was similar to those of the two previously reported ligand-free structures of PRs with high-level resistance to DRV (PDB IDs: 4NPU and 3UF3). However, the FS5929R1 PR exhibited a unique curling conformation at the flaps (Figure 3). Because the FS5929R1 PR harbored the major mutation I50V, which was absent in the other two PRs, the unique flap conformation is likely attributable to I50V. The curled flap conformations in the FS5929R1 PR might hinder the DRV access to the active site of dimeric PR because the flap tips tended to curl toward the active site. Furthermore, the unique flap conformation of the FS5929R1 PR would influence DRV resistance through the change in flexibility of the flap in the dimeric PR suggested by our MD simulations (Figure 5) and proposed in previous MD and NMR studies of other PI-resistant mutants (Perryman et al., 2004; Cai et al., 2012). The high mobility of the flaps of the FS5929R1 PR would provide little opportunity for the formation of PI-binding pockets, resulting in reduced opportunity to bind to DRV. Moreover, when the PR binds DRV, the high flap mobility coupled with effects of active site mutations, such as V32I, I47V, and I50V (Kovalevsky et al., 2006; Liu et al., 2008; Mittal et al., 2013), may result in instability of the complex due to the expansion of the PI-binding pocket (Cai et al., 2014; Figure 6).

It is also plausible that the preference for the unique conformation in FS5929R1 PR would reduce the PR dimerization inhibition activity of DRV, which is the second inhibitory mechanism of DRV (Koh et al., 2007, 2011). DRV appears to bind the region around the 32nd, 33rd, 54th, and 82nd residues in monomeric PR (Koh et al., 2011; Huang and Caflisch, 2012; Hayashi et al., 2014). These four residues are positioned close to the flap tips on the FS5929R1 PR structure. Therefore, the curling conformation would also shield these residues, resulting in the inhibition of DRV binding to monomeric PR; in contrast, the presence of four mutations (V32I, L33F, I54V, and V82F) in FS5929R1 PR might directly impair the DRV binding to monomeric PR.

The DRV-resistant FS5929R1 PR remained susceptible to TPV and exhibited low-to-intermediate resistance to ATV and SQV, which was similar to the results of previous reports on distinct DRV-resistant viruses (Dierynck et al., 2007; Saskova et al., 2009; Rhee et al., 2010). Furthermore, the DRV resistance mutation I50V was related to hypersusceptibility to TPV (Schapiro et al., 2010; Bethell et al., 2012), whereas I50V had the potential to increase susceptibility to ATV (Mittal et al., 2013). Together with our structural information, this information suggests that the distinct susceptibilities might be attributed to the strength of the interactions between the flaps in the PR and these PIs. Crystal structures of the WT PR with TPV, ATV, and SQV (PDB IDs: 1D4Y, 2AQU, and 1HXB, respectively) (Krohn et al., 1991; Thaisrivongs et al., 1996; Clemente et al., 2006) indicate that these PIs commonly have strong interactions with the flaps, which differ from the other PIs. TPV has direct interactions with the main chains of I50 in the flaps, whereas the bulky aromatic rings in ATV and SQV largely interact with the flaps. DRV creates water molecule-mediated hydrogen bonds with the flaps. Therefore, the conformational changes at the flaps may scarcely affect the binding of TPV, ATV, and SQV but not DRV. Obtaining information on the interactions between PR and PIs will aid in the development of PIs that are more potent than DRV against PRs with accumulated mutations at the flap.

In conclusion, we identified a novel structural feature that influences DRV resistance via *in vitro* selection of HIV-1 variants with high-level resistance to DRV and subsequent determination of a crystal structure and a MD simulation of its PR. The information will aid in the development of potent PIs against DRV-resistant viruses.

## Accession number

Coordinates and structure factors have been deposited into the Protein Data Bank (PDB) with the accession code 5B18.

## Author contributions

Conceived and designed the experiments: WS and YI. Performed the experiments: MN, HO, KS, MF, MMaejima, YK, TM, JH, MMatsuda, AH, and YI. Analyzed the data: MN, HO, KS, MF, MMaejima, and YI. Contributed reagents/materials/analysis tools: YY, AS, NW, WS, and YI. Wrote the paper: MN, HO, and YI.

## Funding

This study was supported by the Japan Society for the Promotion of Science (JSPS) fellows (MN) under grant number 15J12567, by the JSPS (YI) under grant number 15H04740, and by the Japan Agency for Medical Research and Development (AMED) (YI and NW).

### Conflict of interest statement

The authors declare that the research was conducted in the absence of any commercial or financial relationships that could be construed as a potential conflict of interest. The reviewer YTY declared a shared affiliation, though no other collaboration, with one of the authors MF to the handling Editor, who ensured that the process nevertheless met the standards of a fair and objective review.

## Supplementary material

Supplemental Movie S1 **Flap dynamics in WT PR at ligand-free states**.

Supplemental Movie S2 **Flap dynamics in FS5929R1 PR at ligand-free states**.

## References

[B1] Agniswamy J. Shen C. H. Aniana A. Sayer J. M. Louis J. M. Weber I. T. (2012). HIV-1 protease with 20 mutations exhibits extreme resistance to clinical inhibitors through coordinated structural rearrangements. Biochemistry 51, 2819–2828. 10.1021/bi2018317 22404139 PMC3328860

[B2] Bethell R. Scherer J. Witvrouw M. Paquet A. Coakley E. Hall D. (2012). Short communication: phenotypic protease inhibitor resistance and cross-resistance in the clinic from 2006 to 2008 and mutational prevalences in HIV from patients with discordant tipranavir and darunavir susceptibility phenotypes. AIDS Res. Hum. Retrovirus. 28, 1019–1024. 10.1089/AID.2011.0242 22098079

[B3] Cai Y. Myint W. Paulsen J. L. Schiffer C. A. Ishima R. Kurt Yilmaz N. (2014). Drug resistance mutations alter dynamics of inhibitor-bound HIV-1 protease. J. Chem. Theory Comput. 10, 3438–3448. 10.1021/ct4010454 25136270 PMC4132871

[B4] Cai Y. Yilmaz N. K. Myint W. Ishima R. Schiffer C. A. (2012). Differential flap dynamics in wild-type and a drug resistant variant of HIV-1 protease revealed by molecular dynamics and NMR relaxation. J. Chem. Theory Comput. 8, 3452–3462. 10.1021/ct300076y 23144597 PMC3491577

[B5] Case D. A. Darden T. A. Cheatham T. E. I. Simmerling C. L. Wang J. Duke R. E. (2006). AMBER 9. San Francisco, CA: University of California.

[B6] Chiba-Mizutani T. Miura H. Matsuda M. Matsuda Z. Yokomaku Y. Miyauchi K.. (2007). Use of new T-cell-based cell lines expressing two luciferase reporters for accurately evaluating susceptibility to anti-human immunodeficiency virus type 1 drugs. J. Clin. Microbiol. 45, 477–487. 10.1128/JCM.01708-06 17182760 PMC1829063

[B7] Clemente J. C. Coman R. M. Thiaville M. M. Janka L. K. Jeung J. A. Nukoolkarn S.. (2006). Analysis of HIV-1 CRF_01 A/E protease inhibitor resistance: structural determinants for maintaining sensitivity and developing resistance to atazanavir. Biochemistry 45, 5468–5477. 10.1021/bi051886s 16634628 PMC2518317

[B8] Delaugerre C. Pavie J. Palmer P. Ghosn J. Blanche S. Roudiere L.. (2008). Pattern and impact of emerging resistance mutations in treatment experienced patients failing darunavir-containing regimen. AIDS 22, 1809–1813. 10.1097/QAD.0b013e328307f24a 18690163

[B9] De Meyer S. Azijn H. Surleraux D. Jochmans D. Tahri A. Pauwels R.. (2005). TMC114, a novel human immunodeficiency virus type 1 protease inhibitor active against protease inhibitor-resistant viruses, including a broad range of clinical isolates. Antimicrob. Agents Chemother. 49, 2314–2321. 10.1128/AAC.49.6.2314-2321.2005 15917527 PMC1140553

[B10] De Meyer S. Lefebvre E. Azjin H. de Baere I. van Baelen B. de Bethune M. P. (2006). Phenotypic and genotypic determinants of resistance to TMC 114: pooled analysis of POWER 1, 2 and 3, in 15th International HIV Drug Resistance Workshop (Sitges).

[B11] Deng N. J. Zheng W. Gallicchio E. Levy R. M. (2011). Insights into the dynamics of HIV-1 protease: a kinetic network model constructed from atomistic simulations. J. Am. Chem. Soc. 133, 9387–9394. 10.1021/ja2008032 21561098 PMC3116037

[B12] Dierynck I. de Wit M. Gustin E. Keuleers I. Vandersmissen J. Hallenberger S.. (2007). Binding kinetics of darunavir to human immunodeficiency virus type 1 protease explain the potent antiviral activity and high genetic barrier. J. Virol. 81, 13845–13851. 10.1128/JVI.01184-07 17928344 PMC2168871

[B13] Dierynck I. van Marck H. van Ginderen M. Jonckers T. H. Nalam M. N. Schiffer C. A.. (2011). TMC310911, a novel human immunodeficiency virus type 1 protease inhibitor, shows *in vitro* an improved resistance profile and higher genetic barrier to resistance compared with current protease inhibitors. Antimicrob. Agents Chemother. 55, 5723–5731. 10.1128/AAC.00748-11 21896904 PMC3232804

[B14] Emsley P. Lohkamp B. Scott W. G. Cowtan K. (2010). Features and development of Coot. Acta Crystallogr. D Biol. Crystallogr. 66, 486–501. 10.1107/S0907444910007493 20383002 PMC2852313

[B15] Ghosh A. K. Sridhar P. R. Leshchenko S. Hussain A. K. Li J. Kovalevsky A. Y.. (2006). Structure-based design of novel HIV-1 protease inhibitors to combat drug resistance. J. Med. Chem. 49, 5252–5261. 10.1021/jm060561m 16913714

[B16] Hattori J. Shiino T. Gatanaga H. Yoshida S. Watanabe D. Minami R.. (2010). Trends in transmitted drug-resistant HIV-1 and demographic characteristics of newly diagnosed patients: nationwide surveillance from 2003 to 2008 in Japan. Antiviral Res. 88, 72–79. 10.1016/j.antiviral.2010.07.008 20692295

[B17] Hayashi H. Takamune N. Nirasawa T. Aoki M. Morishita Y. Das D.. (2014). Dimerization of HIV-1 protease occurs through two steps relating to the mechanism of protease dimerization inhibition by darunavir. Proc. Natl. Acad. Sci. U.S.A. 111, 12234–12239. 10.1073/pnas.1400027111 25092296 PMC4142999

[B18] Heaslet H. Rosenfeld R. Giffin M. Lin Y. C. Tam K. Torbett B. E.. (2007). Conformational flexibility in the flap domains of ligand-free HIV protease. Acta Crystallogr. D Biol. Crystallogr. 63, 866–875. 10.1107/S0907444907029125 17642513

[B19] Hornak V. Abel R. Okur A. Strockbine B. Roitberg A. Simmerling C. (2006a). Comparison of multiple Amber force fields and development of improved protein backbone parameters. Proteins 65, 712–725. 10.1002/prot.21123 16981200 PMC4805110

[B20] Hornak V. Okur A. Rizzo R. C. Simmerling C. (2006b). HIV-1 protease flaps spontaneously open and reclose in molecular dynamics simulations. Proc. Natl. Acad. Sci. U.S.A. 103, 915–920. 10.1073/pnas.0508452103 16418268 PMC1347991

[B21] Huang D. Caflisch A. (2012). How does darunavir prevent HIV-1 protease dimerization? J. Chem. Theory Comput. 8, 1786–1794. 10.1021/ct300032r 26593669

[B22] King N. M. Melnick L. Prabu-Jeyabalan M. Nalivaika E. A. Yang S. S. Gao Y.. (2002). Lack of synergy for inhibitors targeting a multi-drug-resistant HIV-1 protease. Protein Sci. 11, 418–429. 10.1110/ps.25502 11790852 PMC2373441

[B23] Koh Y. Amano M. Towata T. Danish M. Leshchenko-Yashchuk S. Das D.. (2010). *In vitro* selection of highly darunavir-resistant and replication-competent HIV-1 variants by using a mixture of clinical HIV-1 isolates resistant to multiple conventional protease inhibitors. J. Virol. 84, 11961–11969. 10.1128/JVI.00967-10 20810732 PMC2977898

[B24] Koh Y. Aoki M. Danish M. L. Aoki-Ogata H. Amano M. Das D.. (2011). Loss of protease dimerization inhibition activity of darunavir is associated with the acquisition of resistance to darunavir by HIV-1. J. Virol. 85, 10079–10089. 10.1128/JVI.05121-11 21813613 PMC3196396

[B25] Koh Y. Matsumi S. Das D. Amano M. Davis D. A. Li J.. (2007). Potent inhibition of HIV-1 replication by novel non-peptidyl small molecule inhibitors of protease dimerization. J. Biol. Chem. 282, 28709–28720. 10.1074/jbc.M703938200 17635930

[B26] Koh Y. Nakata H. Maeda K. Ogata H. Bilcer G. Devasamudram T.. (2003). Novel bis-tetrahydrofuranylurethane-containing nonpeptidic protease inhibitor (PI) UIC-94017 (TMC114) with potent activity against multi-PI-resistant human immunodeficiency virus *in vitro*. Antimicrob. Agents Chemother. 47, 3123–3129. 10.1128/AAC.47.10.3123-3129.2003 14506019 PMC201142

[B27] Kovalevsky A. Y. Tie Y. Liu F. Boross P. I. Wang Y. F. Leshchenko S.. (2006). Effectiveness of nonpeptide clinical inhibitor TMC-114 on HIV-1 protease with highly drug resistant mutations D30N, I50V, and L90M. J. Med. Chem. 49, 1379–1387. 10.1021/jm050943c 16480273 PMC3015180

[B28] Krohn A. Redshaw S. Ritchie J. C. Graves B. J. Hatada M. H. (1991). Novel binding mode of highly potent HIV-proteinase inhibitors incorporating the (R)-hydroxyethylamine isostere. J. Med. Chem. 34, 3340–3342. 1956054 10.1021/jm00115a028

[B29] Lam P. Y. Jadhav P. K. Eyermann C. J. Hodge C. N. Ru Y. Bacheler L. T.. (1994). Rational design of potent, bioavailable, nonpeptide cyclic ureas as HIV protease inhibitors. Science 263, 380–384. 8278812 10.1126/science.8278812

[B30] Liu F. Boross P. I. Wang Y. F. Tozser J. Louis J. M. Harrison R. W.. (2005). Kinetic, stability, and structural changes in high-resolution crystal structures of HIV-1 protease with drug-resistant mutations L24I, I50V, and G73S. J. Mol. Biol. 354, 789–800. 10.1016/j.jmb.2005.09.095 16277992 PMC1403828

[B31] Liu F. Kovalevsky A. Y. Tie Y. Ghosh A. K. Harrison R. W. Weber I. T. (2008). Effect of flap mutations on structure of HIV-1 protease and inhibition by saquinavir and darunavir. J. Mol. Biol. 381, 102–115. 10.1016/j.jmb.2008.05.062 18597780 PMC2754059

[B32] Maguire M. F. Guinea R. Griffin P. MacManus S. Elston R. C. Wolfram J.. (2002). Changes in human immunodeficiency virus type 1 Gag at positions L449 and P453 are linked to I50V protease mutants *in vivo* and cause reduction of sensitivity to amprenavir and improved viral fitness *in vitro*. J. Virol. 76, 7398–7406. 10.1128/JVI.76.15.7398-7406.2002 12097552 PMC136352

[B33] Martin P. Vickrey J. F. Proteasa G. Jimenez Y. L. Wawrzak Z. Winters M. A.. (2005). “Wide-open” 1.3 A structure of a multidrug-resistant HIV-1 protease as a drug target. Structure 13, 1887–1895. 10.1016/j.str.2005.11.005 16338417

[B34] Mittal S. Bandaranayake R. M. King N. M. Prabu-Jeyabalan M. Nalam M. N. Nalivaika E. A.. (2013). Structural and thermodynamic basis of amprenavir/darunavir and atazanavir resistance in HIV-1 protease with mutations at residue 50. J. Virol. 87, 4176–4184. 10.1128/JVI.03486-12 23365446 PMC3624360

[B35] Murshudov G. N. Vagin A. A. Dodson E. J. (1997). Refinement of macromolecular structures by the maximum-likelihood method. Acta Crystallogr. D Biol. Crystallogr. 53, 240–255. 10.1107/S0907444996012255 15299926

[B36] Ode H. Matsuyama S. Hata M. Hoshino T. Kakizawa J. Sugiura W. (2007a). Mechanism of drug resistance due to N88S in CRF01_AE HIV-1 protease, analyzed by molecular dynamics simulations. J. Med. Chem. 50, 1768–1777. 10.1021/jm061158i 17367119

[B37] Ode H. Matsuyama S. Hata M. Neya S. Kakizawa J. Sugiura W.. (2007b). Computational characterization of structural role of the non-active site mutation M36I of human immunodeficiency virus type 1 protease. J. Mol. Biol. 370, 598–607. 10.1016/j.jmb.2007.04.081 17524421

[B38] Otwinowski Z. Minor W. (1997). Processing of X-Ray diffraction data collected in oscillation mode. Meth. Enzymol. 276, 307–326. 10.1016/S0076-6879(97)76066-X 27754618

[B39] Pazhanisamy S. Stuver C. M. Cullinan A. B. Margolin N. Rao B. G. Livingston D. J. (1996). Kinetic characterization of human immunodeficiency virus type-1 protease-resistant variants. J. Biol. Chem. 271, 17979–17985. 8663409 10.1074/jbc.271.30.17979

[B40] Perryman A. L. Lin J. H. McCammon J. A. (2004). HIV-1 protease molecular dynamics of a wild-type and of the V82F/I84V mutant: possible contributions to drug resistance and a potential new target site for drugs. Protein Sci. 13, 1108–1123. 10.1110/ps.03468904 15044738 PMC2280056

[B41] Prabu-Jeyabalan M. Nalivaika E. Schiffer C. A. (2002). Substrate shape determines specificity of recognition for HIV-1 protease: analysis of crystal structures of six substrate complexes. Structure 10, 369–381. 10.1016/S0969-2126(02)00720-7 12005435

[B42] Prado J. G. Wrin T. Beauchaine J. Ruiz L. Petropoulos C. J. Frost S. D.. (2002). Amprenavir-resistant HIV-1 exhibits lopinavir cross-resistance and reduced replication capacity. AIDS 16, 1009–1017. 10.1097/00002030-200205030-00007 11953467

[B43] Rhee S. Y. Taylor J. Fessel W. J. Kaufman D. Towner W. Troia P.. (2010). HIV-1 protease mutations and protease inhibitor cross-resistance. Antimicrob. Agents Chemother. 54, 4253–4261. 10.1128/AAC.00574-10 20660676 PMC2944562

[B44] Saskova K. G. Kozisek M. Rezacova P. Brynda J. Yashina T. Kagan R. M.. (2009). Molecular characterization of clinical isolates of human immunodeficiency virus resistant to the protease inhibitor darunavir. J. Virol. 83, 8810–8818. 10.1128/JVI.00451-09 19535439 PMC2738195

[B45] Schapiro J. M. Scherer J. Boucher C. A. Baxter J. D. Tilke C. Perno C. F.. (2010). Improving the prediction of virological response to tipranavir: the development and validation of a tipranavir-weighted mutation score. Antivir. Ther. 15, 1011–1019. 10.3851/IMP1670 21041916

[B46] Shibata J. Sugiura W. Ode H. Iwatani Y. Sato H. Tsang H.. (2011). Within-host co-evolution of Gag P453L and protease D30N/N88D demonstrates virological advantage in a highly protease inhibitor-exposed HIV-1 case. Antiviral Res. 90, 33–41. 10.1016/j.antiviral.2011.02.004 21338625

[B47] Spinelli S. Liu Q. Z. Alzari P. M. Hirel P. H. Poljak R. J. (1991). The three-dimensional structure of the aspartyl protease from the HIV-1 isolate BRU. Biochimie 73, 1391–1396. 1799632 10.1016/0300-9084(91)90169-2

[B48] Surleraux D. L. Tahri A. Verschueren W. G. Pille G. M. de Kock H. A. Jonckers T. H.. (2005). Discovery and selection of TMC114, a next generation HIV-1 protease inhibitor. J. Med. Chem. 48, 1813–1822. 10.1021/jm049560p 15771427

[B49] Thaisrivongs S. Skulnick H. I. Turner S. R. Strohbach J. W. Tommasi R. A. Johnson P. D.. (1996). Structure-based design of HIV protease inhibitors: sulfonamide-containing 5,6-dihydro-4-hydroxy-2-pyrones as non-peptidic inhibitors. J. Med. Chem. 39, 4349–4353. 10.1021/jm960541s 8893827

[B50] Tremblay C. L. (2008). Combating HIV resistance–focus on darunavir. Ther. Clin. Risk Manag. 4, 759–766. 19209258 10.2147/tcrm.s1709 PMC2621389

[B51] Vargin A. Teplyakov A. (1997). MOLREP: an automated program for molecular replacement. J. Appl. Crystallogr. 30, 1022–1025.

[B52] Wang J. Wolf R. M. Caldwell J. W. Kollman P. A. Case D. A. (2004). Development and testing of a general amber force field. J. Comput. Chem. 25, 1157–1174. 10.1002/jcc.20035 15116359

[B53] Wensing A. M. Calvez V. Gunthard H. F. Johnson V. A. Paredes R. Pillay D.. (2014). 2014 Update of the drug resistance mutations in HIV-1. Top. Antivir. Med. 22, 642–650. 25101529 PMC4392881

[B54] Winn M. D. Ballard C. C. Cowtan K. D. Dodson E. J. Emsley P. Evans P. R.. (2011). Overview of the CCP4 suite and current developments. Acta Crystallogr. D Biol. Crystallogr. 67, 235–242. 10.1107/S0907444910045749 21460441 PMC3069738

[B55] Zhang Y. Chang Y. C. Louis J. M. Wang Y. F. Harrison R. W. Weber I. T. (2014). Structures of darunavir-resistant HIV-1 protease mutant reveal atypical binding of darunavir to wide open flaps. ACS Chem. Biol. 9, 1351–1358. 10.1021/cb4008875 24738918 PMC4076034

